# The effects of velocity-based versus percentage-based resistance training on athletic performances in sport-collegiate female basketball players

**DOI:** 10.3389/fphys.2022.992655

**Published:** 2023-01-10

**Authors:** Mingyang Zhang, Xingyue Liang, Weifeng Huang, Shicong Ding, Guoxing Li, Wei Zhang, Chao Li, Yanfeng Zhou, Jian Sun, Duanying Li

**Affiliations:** ^1^ Digital Physical Training Laboratory, Guangzhou Sport University, Guangzhou, China; ^2^ School of Athletic Training, Guangzhou Sport University, Guangzhou, China; ^3^ Physical Training Institute, Guangzhou Sports Polytechnic, Guangzhou, China

**Keywords:** load prescription, load-velocity relationship, training load monitoring, resistance training, athletic performance, velocity-based resistance training

## Abstract

**Introduction:** The study compared the effects of 6-week (2 sessions/week) velocity-based resistance training (VBRT) and percentage-based resistance training (PBRT) on athletic performance in Sport-College female basketball players.

**Methods:** Fifteen participants were assigned to the VBRT (*n* = 8) or PBRT (*n* = 7) groups. The load in VBRT group were determined through the sessional target velocity and velocity loss monitoring, whereas PBRT group used a fixed-load based on percentage of 1-repetition maximum (1RM). Both groups completed intervention that involved the free weight back squat and bench press using the same relative load (linear periodization from 65% to 95% 1RM). Training loads data was continuously recorded. Measurements at baseline (T0) and post-training (T2) included 1RM, countermovement-jump (CMJ), squat-jump (SJ), eccentric-utilization-ratio (EUR), drop-jump height and reactive-strength-index (DJ, DJ-RSI), plyometric-push-up (PPU), 505 change-of-direction (COD), 10-m、20-m sprint (T-10、T-20), 17 × 15 m drill-lines (17-drill), Hexagon agility, and functional movement screen (FMS). A mid-term (T1) assessment was included to investigate the short-term effects of both methods and the fluctuation of personalized 1RM.

**Results:** No between-group differences were observed at T0 for descriptive variables (*p* > 0.05). Both groups showed significant improvement in strength gains for back squat and bench press, but VBRT showed *likely* t*o very likely* favorable improvements in CMJ, SJ, EUR, DJ-RSI, Hexagon and COD among athletic performance. The VBRT showed *likely* to *very likely* improvements in 17-drill and DJ, while PBRT showed *unclear* effects. The lifted weights adjusted by VBRT method were higher than prescribed by PBRT (*p* < 0.05) for the same subjects.

**Conclusion:** Compared with fixed-load PBRT, VBRT enhanced power and athletic performance despite similar strength gains. VBRT can be regarded as a more functional resistance-training method under linear periodization.

## Introduction

Basketball is considered to be an intermittent high-intensity team sport with high physical demands, mainly based on anaerobic metabolism for energy supply (Castagna et al., 2009). Extensive literature shows that basketball not only has high requirements on teamwork, but is also closely related to individual physical fitness, such as muscle strength (Rice et al., 2017), power (Santos and Janeira, 2012), ability to change directions quickly (Spiteri et al., 2015), agility (Hoffman, 2011), speed and muscular endurance (Kraemer and Ratamess, 2004; Ransone, 2016), all of which are important characteristics of excellent basketball players.

Resistance training (RT) is essential for developing strength and the physical abilities of high-level or elite basketball players (Simenz et al., 2005). In addition to improving athletic performance, effective resistance training can also induce positive adaptations in the nervous system and muscular system, as well as changes in protein content, the number of muscle fibers, and bone density, etc., thereby increasing muscle volume, enhancing muscle strength and explosive strength (Hoffman, 2011). Current evidence suggests that when strength and explosive strength increase after high-intensity resistance training, the rate of force development (RFD), impulse, and efferent neuromuscular driving force increase (Aagaard et al., 2002), thus effectively improving jump performance. Taken together, resistance training has vital and positive effects on basketball players (Chaouachi et al., 2009).

In general, research has demonstrated that periodized RT programs is beneficial to athletes by increasing muscle strength and lowering the risk of injury (Inoue et al., 2015; Caldas et al., 2016; Buskard et al., 2018). However, fluctuations in strength performance, biological variability, and fatigue during prolonged training cycles can affect the daily training status of athletes, which is one of the major problems encountered by strength and conditioning practitioners (González-Badillo et al., 2017). In other words, it is challenging to prescribed the targeted relative load equal to the actual absolute load because acute strength may fluctuate during training and coaches cannot directly measure these changes (Jovanović and Flanagan, 2014; Dorrell et al., 2020). Furthermore, the actual strength of novice players can improve rapidly after only a few training sessions (González-Badillo and Sánchez-Medina, 2010). Based on the changes in performance, some practitioners have made meaningful attempts to develop auto-regulation methods in resistance training (Mann et al., 2010; Helms et al., 2017; Montalvo-Perez et al., 2021). Auto-regulation is a resistance training prescription approach to adjust the training variables intensity, volume and frequency based on the daily individual fluctuations in fitness, fatigue and readiness of the athlete (Larsen et al., 2021). To date the auto-regulation method includes three programs as follow: autoregulatory progressive resistance exercise (APRE, Subjective autoregulation), rating of perceived exertion (RPE, Subjective autoregulation), velocity-based resistance training (VBRT, Objective autoregulation). There is another common one called “ traditional” or " percentage-based resistance training (PBRT, fixed-load)" that requires coaches to individually evaluate each athlete’s 1-repetition maximum value (1RM). The methods mentioned above have gradually become the focus of research in the field of strength training (Zhang et al., 2022). For a long time, the resistance training prescription for physical fitness training to improve muscle performance indicators, such as maximal strength or explosive strength, is mainly to formulate the load and amount of strength training by different percentages of 1RM. Due to its simplicity and practicability, as well as many successful cases, PBRT has long been considered the best strategy for strength training and has been widely used in various sports and different populations (Sander et al., 2013). Therefore, 1RM has been regarded as the gold standard for designing training loads to achieve specific performance goals. However, the approach does not consider the accumulation of life stress that may affect the daily performance of an individual and trainings related fatigue (Mann et al., 2015).

Previous research have demonstrated that a strong correlation between the movement velocity and %1RM during resistance training (Izquierdo et al., 2006). Meanwhile, with the development and popularization of visual monitoring equipment, the use of linear position transducers (LPT) or accelerometer-based technologies to monitor the movement velocity to achieve specific performance goals has resulted in the development of an objective auto-regulation known as velocity-based resistance training. VBRT is a strength training method that prescribes load at a given concentric velocity according to the personalized load velocity profile (LVP) regression equation and completes a certain number of repetitions (Banyard et al., 2018).

VBRT has distinct advantages over other auto-regulation methods. First, VBRT training intervention programs are gradually improved and have positive effects. For example, the target velocity zone can be set to control the load intensity, and the velocity loss threshold can be used to monitor fatigue and training volume. Second, through carefully assessing players’ condition of the day and real-time strength performance, sports injuries caused by overtraining and fatigue may be minimized. Third, VBRT helps determine the optimal velocity and specific load to improve training specificity. Fourth, quantified training kinetics and kinematics output data from VBRT provides immediate auditory feedback to motivate and improve performance (Mann et al., 2015; Weakley et al., 2021).

Recently, several literatures (Peta, 2019) have provided evidence that VBRT is superior to PBRT in terms of strength gain. In an intervention study of 21 rowers by Held et al., VBRT induced greater strength adaptation at lower training-induced stress and volume (Held et al., 2021). Moreover, the study of 28 female soccer players by Ortega et al. (2020) reported that high-speed VBRT was more effective than traditional maximal strength training, and that stimulation generated in squat 1RM, countermovement jump, sprint and muscle mass would lead to better neuromuscular adaptation. Meanwhile, the controlled study from Zhihui (2020) intervening in 20 college students reached the similar results. However, with the increase and deepening of VBRT research, other researchers have reached inconsistent conclusions. For example, Banyard et al. and Orange with his colleagues also conducted back squat training on resistance-trained male (N = 24) and rugby players (N = 27), respectively, and both showed that PBRT was slightly conducive to the improvement of maximal strength. In addition, two studies found no significant difference in the effectiveness between two methods (Peta, 2019; Montalvo-Perez et al., 2021). The number of repetitions from the two methods is also a common research direction for comparison besides the maximal strength. Some recent studies (Banyard et al., 2021; Held et al., 2021) demonstrated that the VBRT method setting velocity loss to monitor training volume in resistance training completed fewer repetitions than PBRT. Despite the importance of these VBRT research, the difference in relative load and absolute load prescriptions between the two methods, particularly due to influence from the changes in individual 1RM, remains unclear. Moreover, no previous studies have assessed the effects of VBRT on the performance of female basketball players.

In this context, the purpose of this study is to compare the effects of VBRT and PBRT on athletic performance in Sport college female basketball players, and to directly compare the load prescription difference under 1RM’s fluctuations between the VBRT and PBRT methods. Recent reviews on VBRT (Liao et al., 2021; Zhang et al., 2022) led us to hypothesize that this approach would induce similar strength gains compared to the PBRT, but would likely result in a greater magnitude of adaptation in other performance tests.

## Materials and methods

### Study design

The present study was a double-blinded, randomized, controlled trial. The study was conducted in accordance with the *Declaration of Helsinki* and was approved by the Ethics Committee of Guangzhou Sport University. Participants volunteered to participate in the study from April 2021 to June 2021. Each participant signed written informed consent after informed of the risks and benefits associated with the study. The study was registered at www.chictr.org.cn (NO. ChiCTR2200056307).

Randomization was performed after the baseline test. Participants were randomized to VBRT or PBRT using poker markers and the poker cards were drawn by an uninformed third party without further participation in the study. Neither the researches nor participants knew which group of intervention subjects would be assigned to receive instruction. The basketball coaches informed the participants that they could not participate in any additional resistance training during the study.

### Participants

**FIGURE 1:**
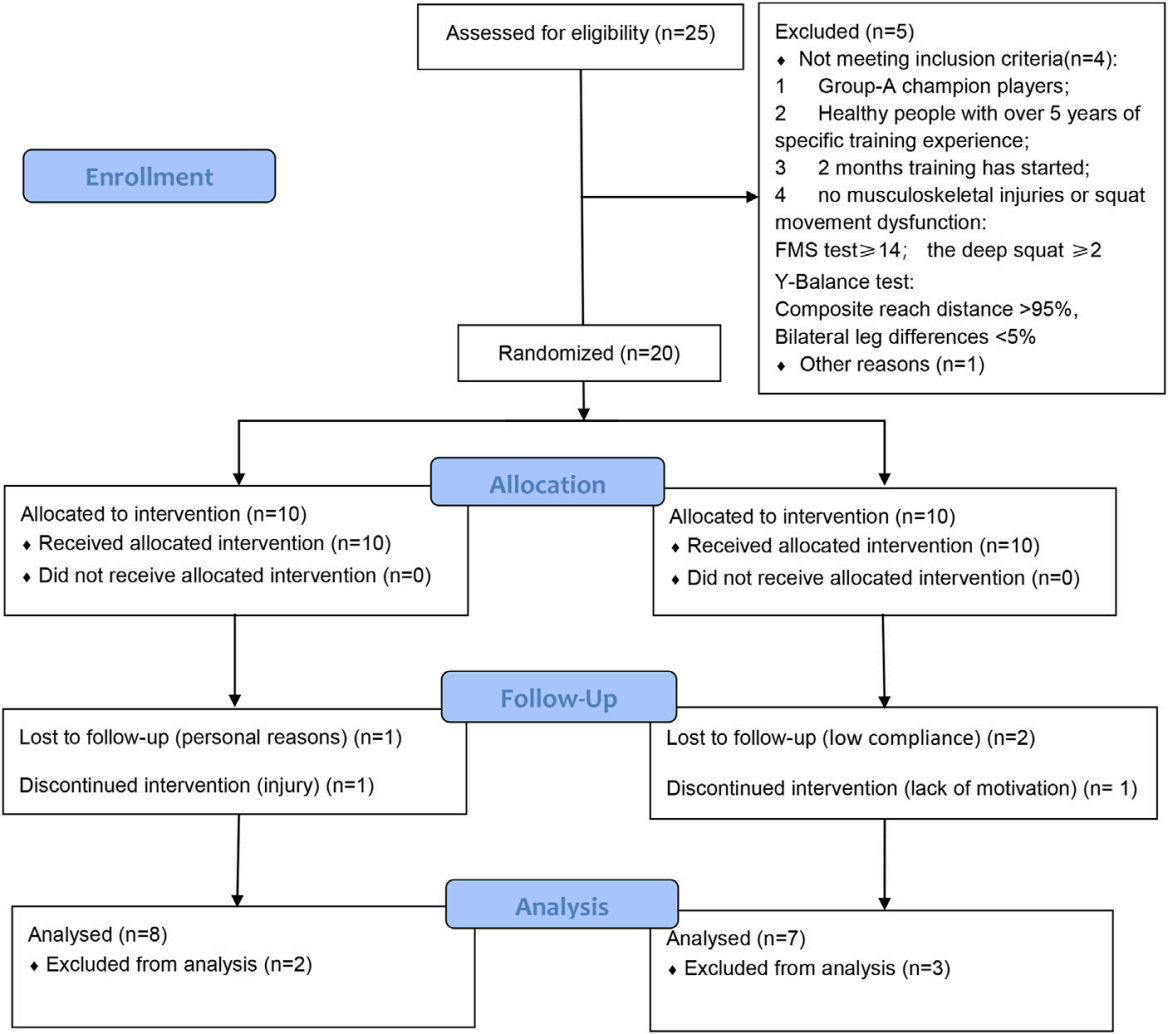
Flowchart for screening, recruitment, allocation, intervention, and follow-up.

The study initially involved 25 sport-collegiate female basketball players voluntarily. Participants were recruited from Guangzhou Sport University basketball team and participated in Sport College Basketball Association (SCBA) Championship. According to the requirements of the experiment, inclusion criteria were no positive case of the functional movement system (there is not pain on completion of movement or screening during FMS and Y-balance), more than five years of basketball-training experience, and no injury in half a year (Figure 1). There were 10 participants were excluded: 5 did not meet the function movement system and 5 dropped out because of injury or illness. Ultimately, 15 participants who met the inclusion criteria were randomly assigned to the VBRT (*n* = 8, age: 22.0 ± 1.2 years; body mass: 59.5 ± 4.4 kg; height: 168.5 ± 6.9 cm) and PBRT (*n* = 7, age: 21.7 ± 2.3 years; body mass: 60.4 ± 7.0 kg; height: 169.0 ± 7.4 cm). During the 6-week intervention, no medical issues or musculoskeletal injuries that may interfere with training were identified, and none of the participants used any medications or dietary supplements. Moreover, participants completed two basketball training sessions and one weight training session each week in the two months before the research. They were asked to perform resistance training with proper technique involved the back squat and bench press, as part of their conditioning training.

### Experimental design

Based on the progressive linear periodization structure (from 60% to 95% 1RM) (Bompa and Buzzichelli, 2019), the RT program (Table 2) was utilized to compare the effects of VBRT and PBRT on muscle strength and power adaptation of upper and lower limb. From April 2021 to June 2021, each participants completed resistance training sessions for 6 weeks (2 sessions/week on Mondays and Wednesdays). Training goals, targeted relative loads, number of sets, and interval were equal but different absolute loads (lifted weight: weight lifted by the individual during each training session) and repetitions between two groups. In addition to the main exercises (back squat and bench press), the same supplementary exercises (Romanian deadlift/Nordic lower, chin-up, and front plank) using a body weight to adjust load and the same volume. One week before the baseline test, the subjects started to get familiar with the test procedures and rating of perceived exertion (RPE) scale (Robertson et al., 2003).

Group velocity zones for each resistance training were created using data collected within the baseline 1RM assessments. The target mean concentric velocity (MCV) of squat (MCV = 0.8–0.38 m⋅s^−1^) and bench press (MCV = 0.8–0.32 m⋅s^−1^) were the target velocity zone from the VBRT (González-Badillo et al., 2011) while 65–95%1RM of squat and of bench press were the goals of the PBRT. During the periodization, the differences between the two groups was the number of repetitions and the actual lifted weight as prescribed by two methods (auto-regulation and fixed-load). One researcher from each group provided technical supervision and protection during training. The subjects were instructed to complete the concentric phase with maximum effort. The strong verbal encouragements were given to participants in the VBRT and PBRT.

### Resistance training program

#### Percentage-based resistance training

The PBRT group performed RT exercises with fixed-load (intensity and repetitions) from 65 to 95% of baseline 1RM (Table 2). These absolute loads were regularly prescribed in the RT program, following the periodization of load (increase in intensity and decrease in volume) (Orange et al., 2019). The training loads were not adjusted within session during the 6-week mesocycle.

#### Velocity-based resistance training

Training monitoring and adjustable loads were integrated into the VBRT program. The lifted weight was prescribed using the target velocity zones, and the number of repetitions was monitored by velocity loss (Jovanović and Flanagan, 2014). During each session, the lifted weight was based on the MCV in relation to the predetermined velocity zone and could be adjusted according to the MCV of the preceding set’s repetitions. Thereafter, previous studies (Orange et al., 2020) have shown that, if the MCV in a set was ±0.06 m⋅s^−1^ outside of the sessional target velocity, the lifted weight was then adjusted by ±5% 1RM for the subsequent set between the sessions for back squat and bench press. MCV monitoring was used to dictate intensity autoregulation (lifted weight) and volume autoregulation (number of repetitions), on real-time, set-by-set basis.

During each training session, the researcher assessed the average barbell velocity (transmitted from the linear position transducer to the iPad *via* Bluetooth) and made appropriate load adjustments for each participant involved in the VBRT. Training data (weight and repetitions) and kinetics data were collected. Throughout the intervention, all participants used a 20 kg barbell and members of the VBRT group used 4 linear position transducers (GymAware Power Tool; Power Performance Technology, Australia), mounted 60 cm right of the center of the barbell, to collect MCV data for each section of the back squat and bench press. All participants were divided into 2-3 a group and were asked to perform back squat and bench press in sequence. To ensure consistency between groups, participants took turns performing a single set of back squat (or bench press) training, with the interval of 90s∼3 min depending on the intensity during the periodization (Table 2).

### Testing procedures

**FIGURE 2:**
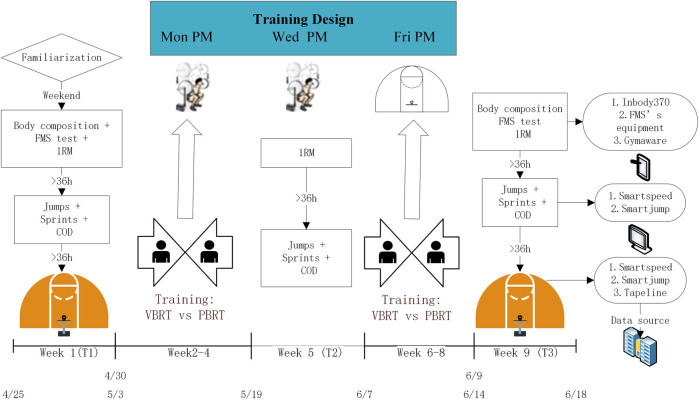
Overall experimental study design and weekly off-season training schedule during the 6-week resistance training.

The outcomes were assessed at baseline (T0), middle-test (T1), and post-test (T2). Testing consisted of ① 1RM strength assessments, including back squat and bench press, ② explosive strength ③ specific performance (no T1) (described in Figure 2). Among them, specific athletic performance was assessed only at T1 and T2. Athletic performance analysis were regarded as the primary outcome and secondary outcome, respectively. Before all tests, participants performed the standardized warm-up (including an easy pace jog, dynamic stretch and lower limb joint activation exercises).

Participants completed all tests on three different sessions, with more than 45 h of recovery between each session. The tests were carried out at the similar time (±1 h) and in the same venue (physical training laboratory and basketball court), with similar environmental conditions (∼28°C and ∼68% of humidity). All tests were supervised and verbally encouraged by expert instructors present in order to play at the highest level.

### Outcome measures

#### Training loads

During weekly training sessions, the individual lifted weights for both methods (VBRT and PBRT) were recorded. In addition, to comprehensively reflect the difference in prescription intensity between the two methods, the %1RM prescription load intensity (VBRT-PB) from participants in the VBRT group was also included for comparison. VBRT-PB is a hypothetical load that calculated from percentage-based (%1RM) in the same subjects of VBRT group, which is compared with the actual lifted weight adjusted by MCV.

#### Functional movement screen (FMS)

The FMS was used to assess different fundamental movement patterns (Kraus et al., 2014). The FMS was carried out before the standardized warm-up, with 7 movements (including the deep squat, hurdle step, in-line lunge, shoulder mobility, straight leg raise, trunk stability push-up, and quadruped rotary stability) and some clearing tests screened by two FMS Level 2 testers. The deep squat score, positive cases, and total scores were combined to assess the athletes’ basic movement ability (flexibility and stability), which was defined as the inclusion criteria for this study. Participants performed the screens without a standardized warm-up. In addition to being an inclusion criterion, the deep squat, straight leg raise, and trunk stability push-up were also considered as test variable for further analysis.

All participants completed 3 times each component test, and the best score achieved was recorded. The scores of all movements were summed, resulting in a composite score from 0 to 21 points, with 21 being the maximum composite score and 14 being the minimum score (<14 will be excluded).

#### Y-balance test (YBT)

The YBT assessed balance while participants reached in the anterior, posteromedial, and posterolateral directions, which was used to evaluate dynamic balance ability, functional symmetry, and neuromuscular control to predict injury risk of the lower limb by comparing the difference between the farthest distance reached by the right and left limbs in each direction and the magnitude of the combined value (Gribble et al., 2012). Participants were assessed in the laboratory for YBT using Y Balance Test Kit™ (Move2Perform, Evansville, IN, USA) and their limb length in supine lying (anterior superior iliac spine to ipsilateral medial ankle center) was measured. The participants were asked to barefoot standing on the platform with the thumb aligned to the red starting line. One foot pushed the test board as far as they could and recorded the maximum reach distance (closest to 0.5 cm) of pushing the test board in different directions, and repeated three times. The test was repeated on the other foot and the results were recorded. If the bilateral difference was >5%, suggesting a significant difference in strength or balance between the left and right side of the support leg, and it was considered a positive case and should be excluded from this study. Test-retest reliability ranged from 0.80 to 0.93 for YBT. The intraclass correlation coefficient (ICC) and coefficient of variation (CV) were observed at baseline in anterior (ICC = 0.93, 95%CI: 0.87–0.96, CV = 2.73%), posteromedial (ICC = 0.80, 95%CI: 0.66–0.97, CV = 3.36%), and posterolateral (ICC = 0.90, 95%CI: 0.81–0.95, CV = 1.44%).

### Muscle strength

The 20 kg barbells were used, with linear position transducer (GymAware Power Tool; Kinetic Performance Technologies, Canberra, Australian Capital Territory, Australia) mounted 60 cm to the right of the center of the barbell, allowing the MCV to be calculated. The same standardized warm-up exercises (included a set of 8–10 repetitions with the empty bar) were performed for both test and participants started 1RM assessments, including ≤50% 1RM (5 repetitions), 70% 1RM (3-5 repetitions), 80% 1RM (2 repetitions) and 90% of 1RM (1 repetition). The participants then attempted to increase the load of 1RM. The last successful lift reaching a parallel depth squat (thighs parallel to the floor), which was supervised by two researchers and GymAware software, being considered as the baseline 1RM. A rest period was given between the sub-maximal set (2 min) and the 1RM attempt (3 min), and a failed 1RM lifted could be attempted again in 5 min, allowing a maximum of 2 attempts. Each participant was given four to six attempts. For all repetitions, participants were required to perform a controlled eccentric velocity and make maximal efforts during the concentric phase. Both groups used the same squat technique throughout the study (included warm-up, training and test).

For the bench press, the participants should perform an adequate warm-up with 5–10 reps of a light-to-moderate weight, then after a minute rest perform two heavier warm-up sets of 2-5 reps, with a two-minute rest between sets. The subject should rest for two to four minutes, then perform the 1RM attempt with the proper technique. If the lift was successful, rest for another two to four minutes and increased the load by 5–10%, and attempt another lift. If the subject failed to perform the lift with the correct technique, rest for two to four minutes and attempted a weight 2.5–5% lower. Keep increasing and decreasing the weight until 1RM was achieved.

### Jump test and plyometric push-up test

Countermovement jump (CMJ test): During each attempt, squat down to the optimal personal depth at the best eccentric speed, and then immediately move vertically up quickly in order to achieve maximum vertical height.

#### Squat jump (SJ test)

Under the guidance of the researchers, the athletes must lower their bodies to the semi-squat position (≈90° knee flexion as monitored by the researcher) and stay still for 2 s. After a two-second pause, the players jumped as high as possible without any countermovement.

#### Drop jump (DJ test)

Participants were required to stand on jump box (40 cm height) behind the jump mat. Under the guidance of the researchers, participants stepped off from the jump box and drop onto the jump mat, then immediately propel back up into a jump.

Participants were asked to keep their hips, ankles, and knees straight throughout the flying phase while putting their hands on their hips. They were also instructed to try to land in the same position as when they took off. Any deviation would result in failure. Each jump test was performed three times, with 30–45 s rest between jumps. Jump tests were measured using a mobile contact mat (Smart Jump; Fusion Sport, Queensland, Australia). The highest height (in centimeters) of all jumps and reactive strength index of drop jump (RSI-DJ) were used for further analysis.

#### Eccentric utilization ratio (EUR) and stretch-shortening cycle % difference (SSC%)

EUR and SSC% could assess the efficiency of energy and power transfer during the subjects’ jumps (Doyle, 2005). Both parameters helped coaches in identifying deficiencies in subjects’ jump performance, allowing them to design more optimized and improved training programs. EUR and SSC% were calculated using the following equation:

EUR=CMJs/SJs

$$SSC\%=\left(CMJs-SJs\right)/CMJs$$

All plyometric forms are based on the Stretch-Shortening Cycle. It is composed of three forms of muscle contraction: eccentric, isometric, and concentric. SSC% aims to quantify the efficiency of SCC. This assessment uses the percentage difference between CMJ and SJ (Hawkins et al., 2009).

#### Standing long jump (SLJ)

The tape and commercial Long Jump Landing Mats were both available for measuring the distance. The participants stood behind a line marked on the ground with feet slightly apart. A two-foot take-off and landing were used, with the swinging of the arms and bending of the knees to provide forward drive. The subjects attempted to jump as far as possible, landing on both feet without falling backward. Three attempts were permitted, with the longest trial being selected for further data analysis.

#### Plyometric push-up test (PPU test)

The participants began in a push-up position, dropped to around 90° of the elbow joints at the appropriate speed, and then instantly pushed up, maintaining their hands straight in the flight phase and landing in the same position as the preparation phase. Three times were completed with 20-s intervals. PPU test were performed, with the highest height (in centimeters) and relative peak power (RPP) used for further analysis.

RPP=push peak power/ body mass

The test-retest reliability were observed at baseline in CMJ (ICC = 0.96, 95%CI: 0.93–0.98, CV = 1.65%), SJ (ICC = 0.96, 95%CI: 0.93–0.99; CV = 1.82%), DJ (ICC = 0.89, 95%CI: 0.8–0.97, CV = 2.65%), SLJ (ICC = 0.79, 95%CI: 0.64–0.93, CV = 1.77%), PPU (ICC = 0.79, 95%CI: 0.63–0.94, CV = 6.99%).

### Sprint performance

After the dynamic warm-up and one practice of 20 m running progressive accelerations, two maximum 20 m indoor sprints were performed, with times being recorded at 10 m, 20 m, and 3 min of rest between attempts. The starting positions were standardized, with photocell timing gates placed at the same height at 0, 10 m and 20 m to determine the number of times to cover 0–10 m, 0–20 m (T10, T20). The starting foot was placed after the first gate (Smart Speed, Fusion Sport, Queensland, Australia). Participants were instructed to run as quickly as they could, and the quickest sprint was selected for further analysis. The CV and ICC for T10 were 1.66% and 0.74 (95%CI: 0.55–0.92) and T20 were 0.82% and 0.85 (95% CI: 0.74–0.96).

### 505 COD test

Participants started in a semi-squat position, sprinted 15 m with the maximum effort, and then immediately changed the direction to 180° without touching the ground with their inside hands during the turn. The foot must step on or over the marker line, otherwise, the attempt is considered a failure. Afterward, they needed to sprint 5 m to the starting line (Stewart et al., 2014), with the timing gate (Smart Speed, Fusion Sport, Queensland, Australia) 5 m away from the designated turning point, and the time of 5-m sprint of changing direction was recorded. Subjects completed six COD tests with a 2-min rest between trials, three times with the dominant leg turning off the designated line and three times with non-dominant leg. The fastest trial was used for data analysis. The test-retest reliability was observed during baseline for all participants in COD (ICC = 0.74, 95%CI: 0.57–0.9, CV = 2.1%).

### Hexagon agility test

Participants began at the center of the hexagon, facing the front line, with both feet together. On the instruction “go,” they jumped over the line, then back across the same line into the middle of the hexagon. Afterward, still facing forward with their feet together, they jumped over the next side and back into the hexagon. The participants repeated this process for three full revolutions, keeping their faces forward during the test. They had to face the same direction throughout the test, and their feet could not land on the hexagon’s taped edges or the trial would be stopped and restarted. The best score from two trials is recorded (Sabin and Alexandru, 2015). The test-retest reliability was observed during baseline for all participants in Hexagon (ICC = 0.68, 95%CI: 0.43–0.92, CV = 3.7%).

### Specific speed endurance

**FIGURE 3:**
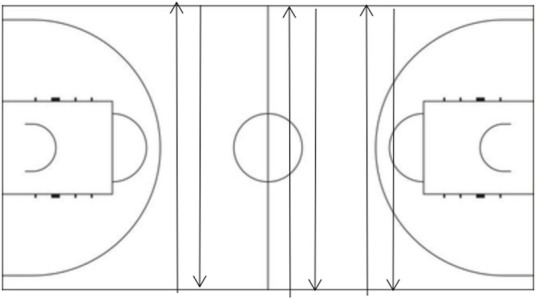
17 × 15 m test.

The 17 × 15 m folding-running (17-drill) assesses the specific speed endurance and ability to change direction multiple times. The participants ran 17 times between the two sidelines of the basketball court for a total of 2 trials, with a 3 min interval between sets (Figure 3). They were divided into two groups (6-8 people each group) for testing, which was monitored by 4 researchers using a stopwatch. Participants were instructed to “step over the edge of the field” each time they turned around throughout the test. The best result was used for analysis. The CV and ICC for 17-drill were 1.88% and 0.67 (95%CI: 0.42–0.91).

### Data collection

The laboratory tests, field tests and session-RPE data were recorded in real-time. All biomechanical data of the MCV and power obtained from the 1RM test or resistance training were collected from the GymAware *via* Bluetooth to a tablet (iPad; Apple Inc., Cupertino, CA) using the app and uploaded onto cloud-based system. The jump height, RPP and DJ-RSI were collected from the Smart Jump online. The sprint performance data of the 10 m, 20 m sprint and 505 COD test was collected from the Smart Speed online.

### Statistical analysis

The mean ± standard deviations (SD) or standardized mean difference (SMD) were calculated using standard statistical methods, and data were then analyzed using the statistical package SPSS (version 21.0, Chicago, IL, USA) and jamovi (version 1.6.23) (Şahin and Aybek, 2019). Test-retest reliability was assessed by CV and ICC with a 95% CI using a one-way random effects model. The reliability was performed using a custom spreadsheet (Hopkins, 2006). The two-sided statistical significance was set at *p* < 0.05 for all tests. Between-group differences in the characteristics of all variables before intervention were examined using an independent sample *t*-test, while intra-group effects in middle test (T0 *vs*. T1) and post-intervention (T0 *vs*. T2) were examined using paired sample *t*-test, with the ESCI package applied to estimate SMD from baseline to postintervention (Şahin and Aybek, 2019). The Shapiro-Wilk test was used to test the normality of all variables, and the variance homogeneity was tested by the Levene test. If the normality was not satisfied, the Mann-Whitney U-tests and Wilcoxon rank tests were performed, expressed as the medians and ranges. A two-factor repeated measures ANOVA 2 (group: VBRT *vs.* PBRT) × 2 (time: T0 *vs*. T2) with Bonferroni *post hoc* test was calculated for each outcome measure and Mauchly’s test was verified for spherical symmetry. Baseline values were included as a covariate for the variables with baseline imbalance (Vickers and Altman, 2001). Inter-group analysis was performed only when a significant group by time interaction was found, with Bonferroni *post hoc* testing used to assess training-related effects. Effect sizes for between-group differences in intervention effects (partial eta squared η_p_^2^: small for 0.01–0.06, moderate for 0.06–0.14, and large for >0.14, respectively) were calculated (Hopkins et al., 2009). The effect size (Hedges’ g, ES) of the within-group differences was calculated for each outcome. To estimate the paired effect sizes between groups, keep in mind that the standardized effect size is SMD, and the standardized effect size has been corrected for bias. The with-group ES were interpreted as trivial (≤0.2), small (0.20–0.60), moderate (0.60–1.20), large (1.20–2.00), or very large (≥2.0) (Hopkins et al., 2009). SMD were additionally calculated as differences between groups (trivial: <0.2, small: 0.2–0.5, moderate: 0.5–0.8, and large ≥0.8) (Cohen, 1988). When there was a significant difference with a null hypothesis, the data was also evaluated for clinical significance using magnitude-based inference (Hopkins et al., 2009). An intervention effect was considered trivial when the mean difference was no more than the smallest worthwhile change (0.2 × between-subject SD). The effect supporting VBRT was reported as positive SMD, whereas the effect supporting PBRT was reported as negative SMD. The qualitative probabilities of beneficial or harmful effects were assessed qualitatively as follows: almost certainly not (<1%); very unlikely (1–5%); unlikely (5–25%); possible (25–75%); likely (75–95%); very likely (95–99%); and almost certain (>99%). If beneficial or harmful change are both >5%, the true difference is assessed as unclear (Hopkins, 2017). Magnitude-based inference approach were calculated using a custom spreadsheet (Hopkins, 2006).

## Results

**FIGURE 4:**
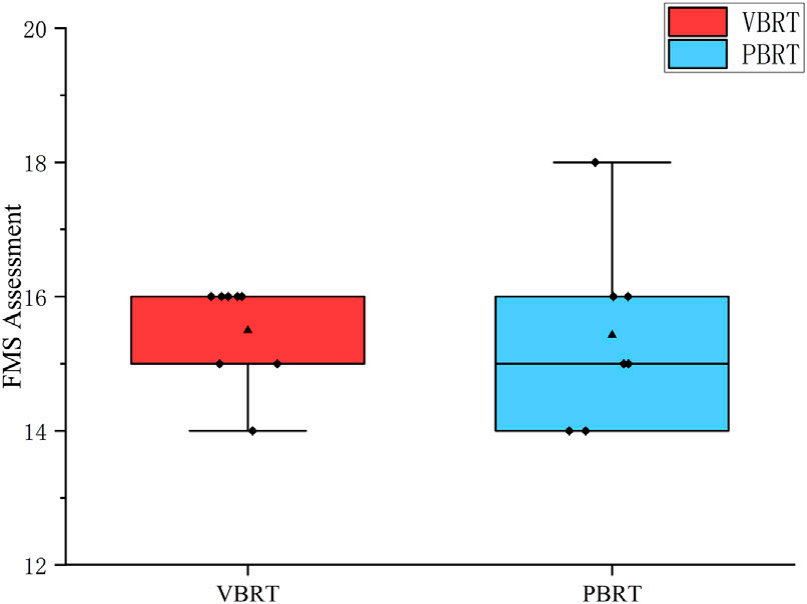
Baseline FMS assessment. FMS test indicates functional movement screen.

Training compliance was 90% in the PBRT group and 86% in the VBRT group throughout the intervention, with no injuries or training-related adverse events. No between-group differences were found at baseline for any variables analyzed in Table 1, Figure 4.

**TABLE 1:** Baseline participant characteristics.

Characteristic	VBRT (N = 8)	PBRT (N = 7)	*p*-value
Age (y) *	22.0 ± 1.2	21.7 ± 2.3	0.76
Height (cm)*	168.5 ± 6.9	169.0 ± 7.4	0.89
BMI *	21.3 ± 1.8	21.4 ± 2.2	0.87
Body mass (kg) *	59.5 ± 4.4	60.4 ± 7.0	0.75
Training years *	8.1 ± 3.2	8.2 ± 2.6	0.85
Back squat 1-RM (kg) *	77.5 ± 12.0	87.1 ± 6.5	0.08
R-SQ 1-RM *	1.3 ± 0.2	1.5 ± 0.2	0.07
Bench press 1-RM (kg) *	37.2 ± 5.4	40.7 ± 4.0	0.18
R-BP 1-RM *	0.63 ± 0.1	0.67 ± 0.1	0.43
FMS test^a^	16 (14–16)	15 (14–16)	0.24
The deep squat^a^	2 (1–3)	2 (1–3)	1

Abbreviations: 1-RM: 1-repetition maximum; BMI: body mass index; R-SQ: relative back squat 1-RM; R-BP: relative bench press 1-RM; FMS test: functional movement screen; VBRT: velocity-based resistance training; PBRT: percentage-based resistance training.

*p*-values for * Independent sample t-test and

^a^ Mann–Whitney U-test were applied to test for differences between the VBRT group and PBRT group.

### Pre-post comparisons

Table 3 presents comprehensive statistical analysis between null-hypothesis significance testing and magnitude-based inference, whereas Figure 8 displays intra- and between-group standardized effects.

### Training load prescription analysis

**FIGURE 5:**
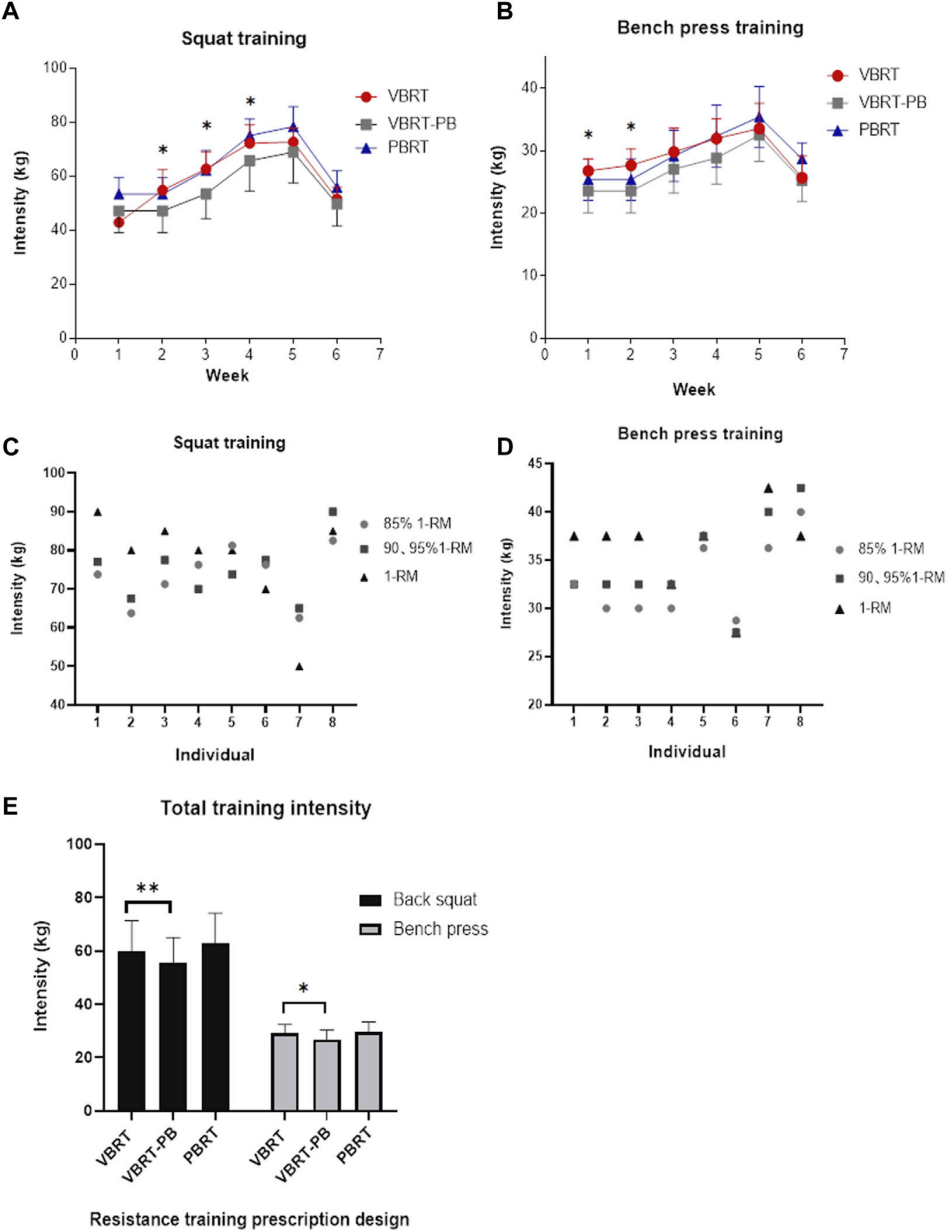
Weekly training load and average weight lifted by VBRT group and PBRT group in back squat **(A)**, or bench press **(B)**, during the heavy load (85∼95%1RM) training sessions, actual weight lifted compared baseline 1RM for different participants in VBRT group in back squat **(C)**, and bench press **(D)**, during linear periodization, average weight lifted from different resistance training prescriptions in back squat or bench press **(E)**. VBRT: velocity-based resistance training; PBRT: baseline %1RM percentage-based resistance training. VBRT-PB: participants in the VBRT group were prescribed load with the %1RM method. *Significant difference VBRT vs. VBRT-PB; #Significant difference VBRT vs. PBRT.

The lifted weight and volume of the training actually performed by the two groups were shown in Table 2. The differences of lifted weight between VBRT and VBRT-PB were shown in Figures 5A, B. The actual lifted weight adjusted by velocity (VBRT) was significantly higher than the load prescribed by percentage-based (VBRT-PB) for the same subjects (59.4 ± 12.3 kg *vs*. 55.3 ± 12.6 kg, respectively; *p* < 0.01, Figure 5E). The number of repetitions from back squat performed each session (32.4 ± 8.7 *vs.* 33.9 ± 12.2 repetitions, Table 2) showed no inter-group difference. For the bench press training, the actual lifted weight was also higher than the load prescribed by percentage-based (29.3 ± 4.2 kg *vs*. 26.8 ± 4.8 kg; *p* < 0.05). Moreover, the number of bench press repetitions in the VBRT group was significantly lower than in the PBRT group (26.3 ± 10.6 *vs*. 30.8 ± 11.9, *p* < 0.01), while weekly strength training sessions showed no difference in RPE between the two training methods (14.4 ± 1.7 *vs*. 14.6 ± 1.4). Interestingly, there were some participants in VBRT who experienced beyond the individual baseline 1RM values load during the 85%1RM and 90, 95%1RM training sessions (Figures 5C, D). Taken together, VBRT induced higher intensity than percentage-based resistance training under the same relative load.

**TABLE 2:** Descriptive characteristics of the resistance training program performed by both experimental groups over the 6-week training period.

Training variable	Week 1		Week 2		Week 3		Week 4		Week 5		Week 6		Total	
Back squat	Light (∼65%1RM)		Light (∼65%1RM)		Moderate (∼75%1RM)		Heavy (∼85%1RM)		Heavy (90–95%1RM)		Light (∼70%1RM)		65%–95%1RM	
	VBRT	PBRT	VBRT	PBRT	VBRT	PBRT	VBRT	PBRT	VBRT	PBRT	VBRT	PBRT	VBRT	PBRT
Intervention	TV + VL: 0.78 ± 0.16	4 × 10-12	TV + VL: 0.75 ± 0.15	4 × 10-12	TV + VL: 0.57 ± 0.06	4 × 8	TV + VL: 0.45 ± 0.05	4×5-6	TV + VL: 0.38 ± 0.04	4×3-4	TV + VL:0.65 ± 0.1	4×6-10	TV: 0.75–0.38	65%–95%1RM
Repetitions	41.3 ± 11.7	47.5 ± 1.8	40.4 ± 8.8	44.6 ± 3.9	35.1 ± 7.73	36.8 ± 4.1	23.4 ± 5.1	21.9 ± 1.9	20.4 ± 3.3	17 ± 2.1*	33.7 ± 4.5	35.8 ± 1.3	32.4 ± 8.7	33.9 ± 12.17
Load (kg)	44.5 ± 4.1	53.3 ± 6.1	54.8 ± 7.6	53.3 ± 6.1	62.7 ± 6.4	62.1 ± 7.4	72.1 ± 6.7	75 ± 6.1	72.6 ± 5.2	78.3 ± 7.4	51.5 ± 4.5	55.8 ± 6.1	59.7 ± 11.9	63 ± 11.9
	VBRT-PB: 47.1 ± 8.1		VBRT-PB: 47.2 ± 8.1		**VBRT-PB: 53.4 ± 9.1#**		VBRT-PB: 65.7 ± 11.3		VBRT-PB: 68.9 ± 11.4		VBRT-PB: 49.6 ± 8.1		VBRT-PB: 55.3 ± 12.6**	
Rest between sets (s)	90–120		90–120		90–120		150		150		90–120		90–150	
Bench press	Light (∼65%1RM)		Light (∼65%1RM)		Moderate (∼75%1RM)		Heavy (∼85%1RM)		Heavy (90–95%1RM)		Light (∼70%1RM)		65%–95%1RM	
Intervention	TV + VL: 0.73 ± 0.15	4 × 10-12	TV + VL: 0.72 ± 0.14	4 × 10-12	TV + VL: 0.54 ± 0.05	4 × 8	TV + VL: 0.42 ± 0.05	4×5-6	TV + VL: 0.35 ± 0.04	4×3-4	TV + VL:0.6 ± 0.06	4×6-8	TV: 0.7–0.35	65%–95%1RM
Repetitions	37.5 ± 9.6	43.3 ± 3.4	36.5 ± 9.3	42.8 ± 4.2	20.3 ± 4.4	28.8 ± 3.3***	16.4 ± 6.3	19.4 ± 1.3	13.4 ± 1.9	14.5 ± 1.1	31.9 ± 5.9	38 ± 5.02	26.3 ± 10.6	30.8 ± 11.9**
Load (kg)	26.8 ± 1.9	25.4 ± 3.3	27.7 ± 2.6	25.4 ± 3.3	29.8 ± 3.9	29.2 ± 4.1	32 ± 3.1	32.3 ± 5	33.6 ± 4.1	35.4 ± 4.9	25.7 ± 3.5	28.7 ± 2.5	29.3 ± 4.2	29.4 ± 5.2
	VBRT-PB: 23.6 ± 3.5*		VBRT-PB: 23.6 ± 3.5*		VBRT-PB:27.1 ± 3.8		VBRT-PB: 28.9 ± 4.2		VBRT-PB: 32.5 ± 4.2		VBRT-PB: 25.3 ± 3.3		**VBRT-PB: 26.8 ± 4.8,#**	
Rest between sets (s)	90–120		90–120		90–120		150		150		90–120		90–150	
RPE	15 ± 1.9	15.7 ± 1.5	14.9 ± 1.8	15 ± 1.7	13.6 ± 0.8	14.2 ± 0.8	15.8 ± 2	14.8 ± 1	15.7 ± 0.5	16 ± 0.9	14.3 ± 2	14.7 ± 1.4	14.4 ± 1.7	14.6 ± 1.4

Abbreviations: %1RM: percentage of 1-repetition maximum; VBRT: velocity-based resistance training that trained with adjustable and variable training loads; PBRT: percentage-based resistance training that trained with fixed training loads; VBRT-PB: participants in the VBRT group were prescribed load with the %1RM method; TV + VL: the target mean concentric velocity and velocity loss; RPE: rating of perceived exertion, the mean RPE of group for each week (2 sessions).

Data are mean ± SD. Statistically significant differences between groups are marked and in bold.: *p < 0.05, **p < 0.01, ***p < 0.001 for Independent sample t-test or #*p* < 0.05 for ^a^Mann–Whitney U-test.

### FMS

No significant within-group differences were observed from T0 to T2.

### Muscle strength

**FIGURE 6:**
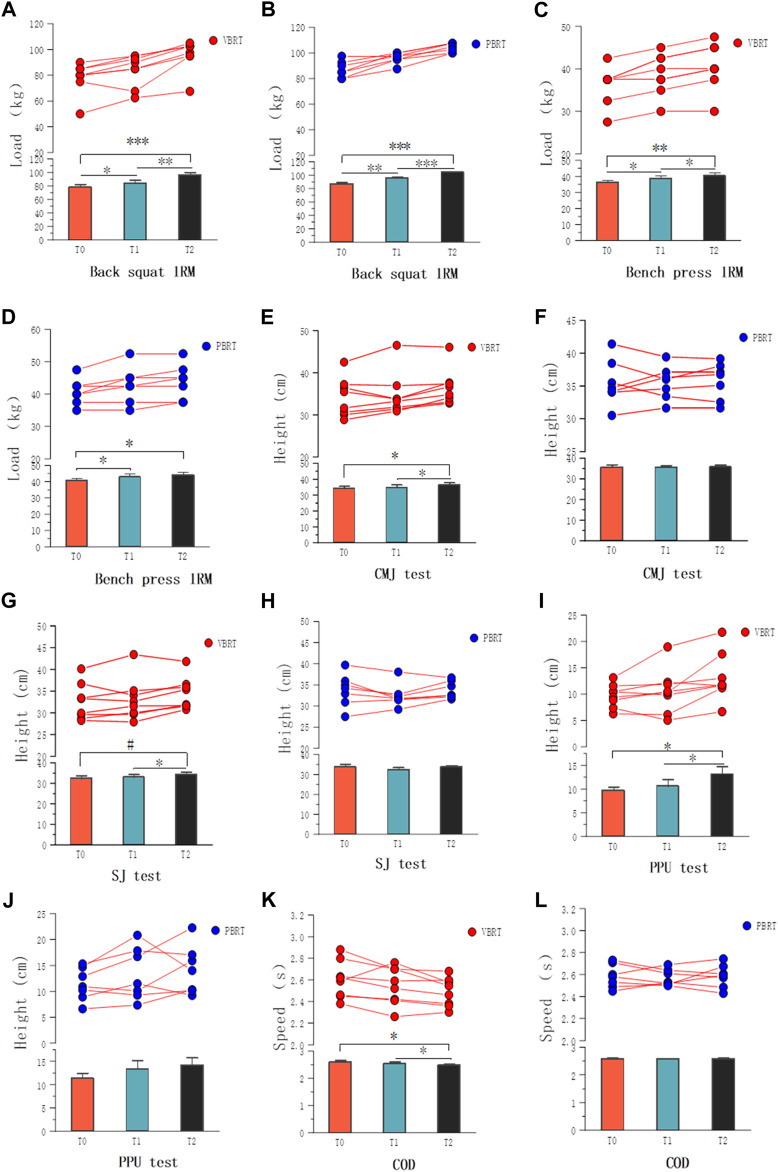
Mean changes in back squat 1RM **(A,B)**, bench press 1RM **(C,D)**, CMJ **(E,F)**, SJ **(G,H)**, PPU **(I,J)** and COD **(K,L)** by group (VBRT-PBRT, respectively) after 3 and 6 weeks of training. *Significant difference in paired t-test; # Significant difference in Wilcoxon rank. RM: repetition maximum; CMJ: countermovement jump; SJ: Static squat jump test; PPU test: plyometric push-up test; COD: 505 change of direction performance; T0: baseline test; T1: intermediate assessments of 3 weeks; T2: post-intervention test.

No significant group by time interaction effects were found for 1RM, but significant time effect was observed for SQ1RM (*p* < 0.001). After the 6-week training intervention, SQ1RM and BP1RM increased significantly in VBRT (squat: *p* < 0.001, ES = 1.39, *almost certainly*; bench press: *p* = 0.008, ES = 0.76, *very likely*) and PBRT (squat: *p* < 0.001, ES = 3.09, *almost certainly*; bench press: *p* = 0.039, ES = 0.63, *likely*) (Figures 6A–D; Table 3).

**TABLE 3:** Effects of the interventions on athletic performance (muscle strength/power and specific performance).

	VBRT							MBI	PBRT							MBI	ANOVA	
	T0	T1	T2	Absolute Δ	*p*-value	ES	SMD		T0	T1	T2	Absolute Δ	*p*-value	ES	SMD		Time effect	Group×time
Maximal strength																		
SQ-1RM (kg)	78.1 ± 12.2	84.1 ± 12.5*	95.9 ± 12.1***	17.8	<.001	1.39	1.38	100/0/0	87.1 ± 6.5	96.1 ± 4.30**	104.3 ± 3.45***	17.1	**<.001**	3.09	3.07	100/0/0	**0.007**	0.761
								Almost certainly ↑								Almost certainly ↑		
BP-1RM (kg)	36.3 ± 4.43	38.8 ± 4.82**	40.3 ± 5.58**	4.06	0.008	0.75	0.76	99.4/0.5/0.1	40.7 ± 4.01	42.9 ± 5.67*	43.9 ± 5.37*	3.21	**0.039**	0.63	0.63	90.9/7.0/2.2	0.709	0.327
								Very likely ↑								Likely		
Power adaptation																		
CMJ (cm)	34.1 ± 4.63	34.8 ± 5.10	36.6 ± 4.28**	2.45	0.003	0.53	0.52	98.6/1.4/0.0	35.8 ± 3.48	35.5 ± 2.55	35.8 ± 2.81	-0.21	0.804	0	-0.06	15.6/57.4/27	**0.017**	**0.04**
								Very likely ↑								Unclear		
SJ (cm)	32.5 ± 4.22	33.1 ± 4.81	34.4 ± 3.74**	1.94	0.016	0.45	0.46	95.1/4.8/0.2	33.7 ± 3.87	32.5 ± 2.71	33.8 ± 2.03	0.04	0.971	0.03	0.01	0/100/0	0.07	0.08
								Very likely ↑								Unclear		
DJ (cm)	33.9 ± 4.62	35.8 ± 4.59**	36.4 ± 4.70*	2.52	0.013	0.51	0.51	96.2/3.7/0.1	33.6 ± 3.25	34.0 ± 3.25	34.6 ± 2.68	1.0O	0.813	0.31	0.31	62/28.3/9.7	**0.015**	0.25
								Very likely ↑								Unclear		
DJ-RSI	0.71 ± 0.19	0.98 ± 0.37**	0.88 ± 0.19*	0.18	0.014	0.85	0.88	97.8/2.0/0.2	0.88 ± 0.26	0.93 ± 0.23	0.96 ± 0.30	0.08	0.123	0.27	0.26	40.3/56.4/3.3	**0.001**	0.34
								Very likely ↑								Unclear		
PPU (cm)	9.7 ± 2.21	10.6 ± 5.0	13.2 ± 4.57*	3.47	0.025	0.92	0.87	97.9/1.4/0.7	11.4 ± 3.13	12.6 ± 5.10	14.1 ± 4.69	2.77	0.086	0.63	0.67	91.7/6.1/2.2	0.564	0.761
								Very likely ↑								Likely		
RPP	22.2 ± 3.32	23.6 ± 3.71	24.2 ± 4.01	2.06	0.102	0.51	0.53	87.8/10.1/2.1	25.7 ± 2.23	25.6 ± 2.5	25.3 ± 4.45	-0.36	0.825	-0.11	-0.1	29.3/17.5/53.2	0.381	0.418
								Possibly								Unclear		
Sprint and COD																		
T-10M (s)	1.93 ± 0.10	1.98 ± 0.10**	1.94 ± 0.08	0.05	0.542	0.1	0.13	30.5/61.6/7.9	1.94 ± 0.05	2.09 ± 0.11**	1.95 ± 0.06	0.02	0.375	0.17	0.29	27/14.7/58.2	0.518	0.561
								Unclear								Unclear		
T-20M (s)	3.55 ± 0.18	3.60 ± 0.20	3.55 ± 0.16	0.02	0.903	0	0.02	40.6/40.6/18.8	3.54 ± 0.08	**3.62 ± 0.15**	3.51 ± 0.13	-0.03	0.639	-0.26	-0.22	51.4/22.4/26.3	0.401	0.599
								Unclear								Unclear		
COD (s)	2.60 ± 0.17	2.55 ± 0.17	2.48 ± 0.13*	-0.11	0.015	-0.87	-0.7	96.9/2.9/0.2	2.58 ± 0.11	2.57 ± 0.07	2.59 ± 0.11	0.002	0.964	0.09	0.02	24.3/27.1/48.6	0.083	0.072
								Very likely ↑								Unclear		
Specific Performance																		
17-drill(s)	69.8 ± 2.4		68.4 ± 1.8*	-1.42	0.046	-0.62	-0.64	92.4/6.9/0.7	69 ± 2.0		68.8 ± 2.4	-0.23	0.801	-0.08	-0.1	43/31.7/25.3	0.092	0.178
								Likely								Unclear		
Hexagon (s)	13.5 ± 1.25		10.6 ± 0.96***	-1.8	<.001	-2.46	-2.47	99.8/0.2/0	13.0 ± 0.73		11.2 ± 0.45**	-2.07	**0.002**	-2.78	-2.83	99.9/0.1/0.1	**<.001**	0.076
								Almost certainly ↑								Almost certainly ↑		
SLJ (cm)	216.5 ± 19.5		219.5 ± 18.3**	2.99	0.002	0.15	0.15	8.2/91.8/0	216.0 ± 9.53		216.2 ± 8.96	0.19	0.848	0.02	0.02	31.9/47.8/20.4	0.155	0.198
								Likely trivial								Unclear		
EUR	1.04 ± 0.03		1.09 ± 0.04*	0.04	0.014	1.34	1.22	98.6/1.0/0.4	1.06 ± 0.04		1.06 ± 0.03	0.00	0.94	0	-0.1	0/99.9/0	0.098	0.052
								Very likely ↑								Unclear		
SSC%	3.98 ± 3.28		8.78 ± 4.14*	4.81	0.014	1.22	1.22	98.7/0.9/0.4	6.37 ± 4.50		5.93 ± 3.41	-0.44	0.94	-0.1	-0.1	37.2/31/31.8	0.098	0.052
								Very likely ↑								Unclear		

Data are mean ± SD., Significances difference (***p < .001; **p < .01; *p < .05) are marked and in bold. *p*-value: Within-group comparison (baseline vs. post-intervention); ES: effect size; SMD, standardized mean difference; MBI: magnitude-based inference analysis.

Abbreviations: T0: baseline assessment before training; T1: end of 3-week training intervention; T2: 6 weeks after end of training; VBRT: velocity-based resistance training; PBRT: %1RM, percentage-based resistance training; SQ-1RM, BP-1RM: one-repetition maximum of back squat or bench press; CMJ, SJ, DJ, SLJ: countermovement jump height, squat jump height, 40 cm drop-jump, standing long jump, respectively; DJ-RSI: the reactive strength of index of drop jump; PPU: plyometric push-up height; RPP: the relative peak power output of plyometric push-up; T-10M, T-20M:10-, 20-m sprint time; COD: 505 change-of-direction test. 17 dirll: 17✖15 m lines-drill test; EUR: eccentric utilization ratio; SSC%: stretch-shortening cycle % difference.

### Jump performance

**FIGURE 7:**
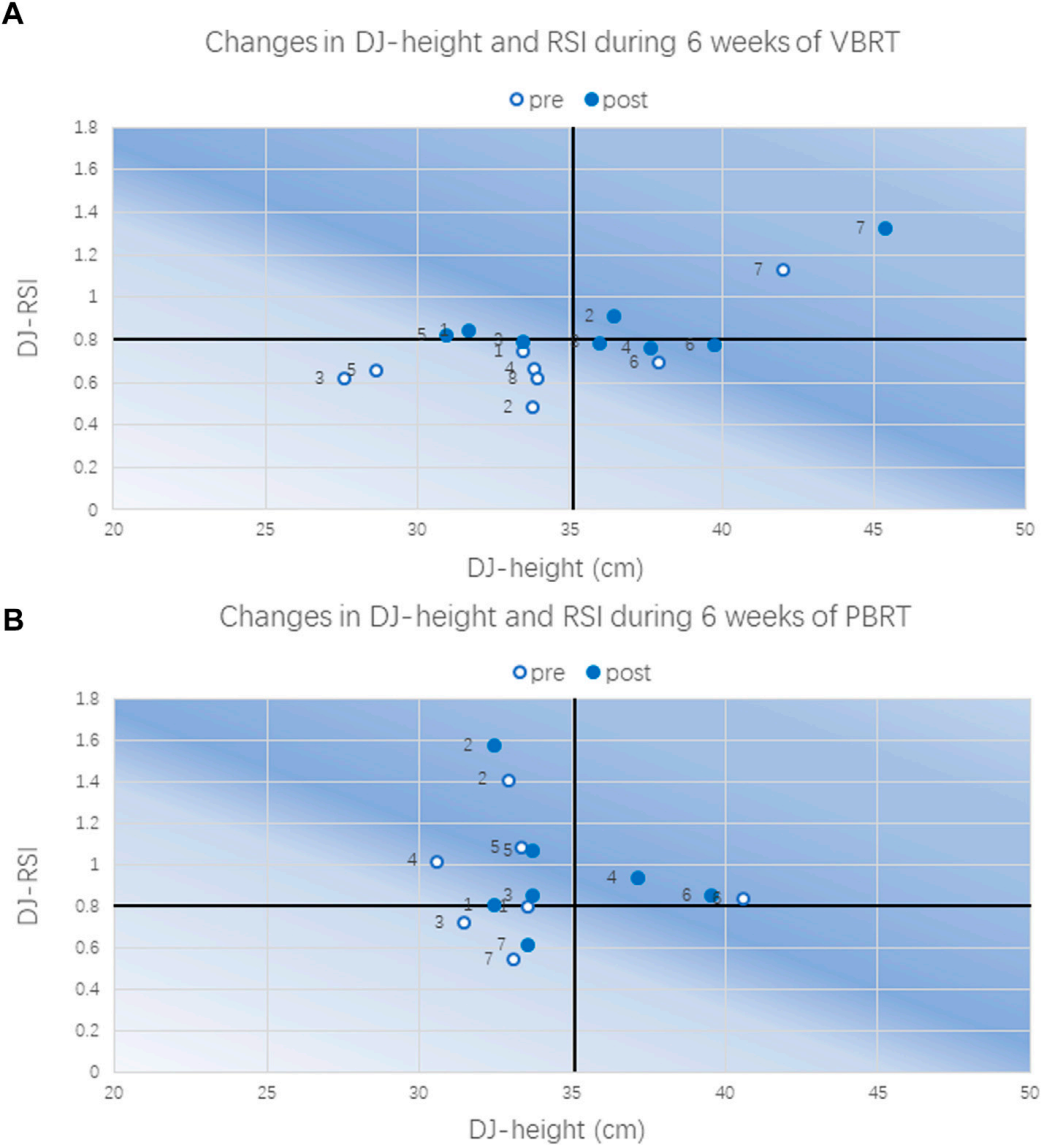
Four-quadrant diagram of changes in DJ-height and RSI in VBRT **(A)** or PBRT **(B)**. DJ-height and DJ-RSI: the height and reactive strength index of 40 cm drop jump test.

**FIGURE 8:**
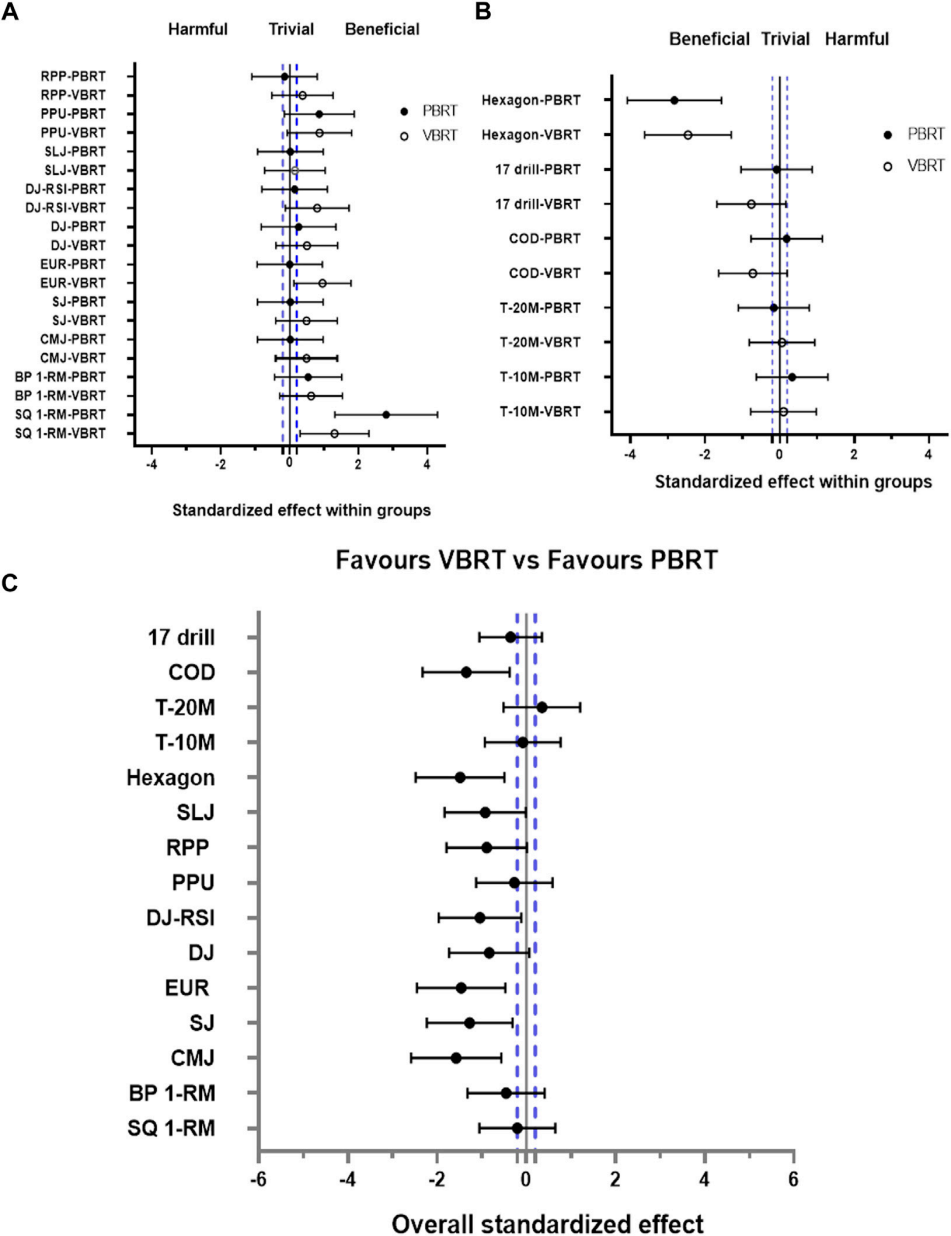
Standardized differences (90% confidence intervals) in all measured variables physical and athletic performance between T0 and T2 for VBRT and PBRT **(A,B)**, and overall standardized differences (90% confidence intervals) in all measured variables between VBRT and PBRT **(C)**. SQ-1RM, BP-1RM: one-repetition maximum of back squat or bench press; CMJ, SJ, DJ, SLJ: countermovement jump height, squat jump, 40 cm-drop jump, standing long jump, respectively; EUR: eccentric utilization ratio; PPU: plyometric push-up height; RPP: the relative peak power output of plyometric push-up, the peak power divided by body mass; Hexagon: hexagon agility test; T-10M, T-20M:10-, 20-m sprint time; COD: 505 change-of-direction test; 17 dirll: 17 × 15 m lines-drill test.

The VBRT and the PBRT showed significant group by time interactions between groups for CMJ height (*p* = 0.04). Significant time effects were observed for CMJ, DJ, DJ-RSI, and PPU-RPP. The CMJ, SJ, and DJ height were increased by 7.8%, 6%, and 7.4% in the VBRT group (*p* < 0.05, ES = 0.53,0.45,0.51, respectively) (Figures 6E, G, I), yet the PBRT group showed no significant within-group improvements in all measured jump variables from T0 to T2 (Figures 6F, H, J). The drop jump performance in the VBRT group improved to the upper right in the four-quadrant diagram from T0 to T2 (Figure 7). Significant within-group improvements was found only in the VBRT group (Table 3). To summarize, the VBRT group improved significantly (*p* < 0.05) in different jump performances (Figure 8A). Furthermore, the VBRT group showed *very likely* beneficial effects with EUR compared to PBRT, while *unclear* effects were observed in the PBRT group (Figure 8A).

### Plyometric push-up test

No significant time effect (*p* > 0.05) and no significant group by time interaction effect was observed for PPU height. After intervention, the VBRT group had a significant improvement for PPU height (*p* < 0.05, ES = 0.92), while the PBRT group showed no significant difference (*p* = 0.086, ES = 0.63).

### Sprint and COD test

No significant group by time interaction and time effect were found in all the results of sprint and COD, and only a significant difference was found in COD (*p* = 0.015, ES = −0.87) in the VBRT (Table 3; Figures 6K, L). When compared to PBRT, the VBRT group may have favorable COD effect (Figure 8). Notably, VBRT and PBRT induced worse effects in T10 and T20 (*p* < 0.05) under the load of 65–75% 1RM from T0 to T1 (primarily muscular hypertrophy and endurance).

### Specific endurance and hexagon test

The significant time effect (*p* < 0.001) was observed in Hexagon test but not in 17-drill. After intervention, the Hexagon test was *almost certainly* improved in both the VBRT and PBRT groups (−14.9% *vs.* −15.6%, *p* < 0.05, ES = −2.46 *vs*. −2.78) (Figure 8). Significant within-group difference from T0 to T2 for 17-drill in the VBRT group.

## Discussion

According to the results, the 6-week VBRT and PBRT methods produced similar overall improvements in muscular strength and agility in female basketball players but did not induce improvements in short sprint speed. Nevertheless, some differences were found between the two methods. The VBRT group was more conducive to inducing the development of various jumping performance, upper limb explosive strength, change of direction ability, reactive strength and specific speed endurance. In addition, this study showed that the actual lifted weight adjusted by velocity was heavier than prescribed by 1RM percentage-based. Under the prescription of 85–95%1RM heavy load, several participants experienced overload beyond the baseline 1RM, indicating a possible benefit mechanism of VBRT that has been missed in previous studies.

### Training loads

Previous studies have investigated the differences in RPE, load, and repetitions between VBRT and PBRT, and three of them have reported that VBRT completed fewer repetitions than PBRT (Peta, 2019; Dorrell et al., 2020; Banyard et al., 2021). The literature above appears to reveal the mechanisms underlying the advantages of VBRT, indicating the ability to enhance training specificity and reduce unnecessary mechanical work to improve motivation and performance (Galiano et al., 2022). In contrast, this study designed a 6-week progressive load, and squat and bench press training were monitored with velocity loss (5–20%). According to the current data, there was no significant difference in RPE between the two groups (14.4 ± 1.7 *vs.* 14.6 ± 1.4). Both back squat and bench press repetitions in VBRT were lower than in PBRT, though only the bench press results were statistically significant (Table 2). The lower volume for similar maximal strength adaptation was a potential advantage worth considering. As for no difference in RPE, it was likely due to the fact that VBRT lifted heavier loads despite fewer repetitions. More detailed studies are needed to confirm the ultimate advantage of VBRT in RPE.

This study compared the real-time lifting load after adjustments with the absolute load (Ruther et al., 1995) from percentage-based (hypothetical load) under the same relative load in the same participants of the VBRT group in addition to the number of repetitions between the VBRT group and the PBRT group. It also compared the lifting load with individual baseline 1RM based on the corresponding movement velocity under the heavy load (back squat 0.38 ± 0.05 m/s, bench press 0.32 ± 0.05 m/s). The above comparative analyses were ignored in previous studies (Pareja-Blanco et al., 2017; Dorrell et al., 2020; Jukic et al., 2022). These results provide support for the hypothesis that the lifted load weight of VBRT was higher than it is designed by percentage-based resistance training. However, these data must be interpreted with caution because no studies have attempted to conduct VBRT mode training under such high intensity. This is mainly attributed to the fact that the maximal strength of athletes is dynamically variable and the individual strength performance state is affected by physiological and psychological factors (González-Badillo and Sánchez-Medina, 2010), with the change or increase of 1RM being ignored (Poliquin, 1988), and thus the prescribed load may not match %1RM for specific training programs (Richens and Cleather, 2014). However, VBRT, which is based on individual MCV and velocity loss to prescribe load intensity and training volume, can be implemented in all aspects of resistance training programming and supports variable prescriptions of load, number of sets, number of repetitions, as well as applied programming methods (de Hoyo et al., 2021). The athletes’ real lifting loads in the VBRT group manifested an overload phenomenon of ≥100%1RM during heavy-load resistance training (Hoffman, 2011). For submaximal load (≤1RM) training, RT was traditionally recommended as a percentage of 1RM (%1RM) or a maximum number of repetitions per set (nRM) (Kraemer and Ratamess, 2004; González-Badillo et al., 2011).

Previous scientific literature (Jovanović and Flanagan, 2014; González-Badillo et al., 2017; Weakley et al., 2020) has shown that fluctuations in strength gains and fatigue are unavoidable over long training cycles, and the obtained baseline frequently do not represent the athlete’s true maximum values (González-Badillo and Sánchez-Medina, 2010). The gains in T1 strength might support the results stated above. It is obvious that the situation is ignored when RT protocol is prescribed using a baseline 1RM. In contrast, the real%1RM can be precisely determined using a velocity-based approach (González-Badillo and Sánchez-Medina, 2010). VBRT can be adjusted for how an individual’s performance is measured (and the perception of potential performance ability) (Greig et al., 2020). The adaptation helps us understand the emergence of mega-intensity (≥1RM), a situation that depends in part on the VBRT adjusting the absolute load based on the participants’ real-time performance after significant improvements in maximal strength, thus allowing individuals to perform resistance training stimuli beyond the 1RM weight. However, with a small sample size, these results must be interpreted with caution. There are still many unanswered questions about VBRT methods. In the past, isometric or eccentric contractions rather than concentric contractions were the major kind of training used with overload resistance (Hoffman, 2011). The idea behind this training technique is to activate the nervous system by supporting weights that are heavier than 100% 1RM (Hoffman, 2011). This training is difficult to carry out because such intensive training involves high risk, and it requires the assistance and real-time monitoring from protectors. Therefore, longer-term research with bigger samples are required to demonstrate the reliability of VBRT, which can provide real-time monitoring for mega-intensity training (≥100% 1RM).

### Muscular strength

The data and the forest plots visually showed that both interventions induced similar maximal strength improvements in the back squat and bench press (*p* < 0.001) (Table 3; Figure 8). Inconsistent findings were revealed in earlier research (Peta, 2019; Dorrell et al., 2020; Held et al., 2021; Orange et al., 2022) investigating maximal strength. The training effects reported in several investigations differ from the current study due to methodological variations (participants, experimental design, and statistical analysis), which can mainly be attributed to methodology differences. Regarding the first discrepancy, it was discovered that VBRT (Zhang et al., 2022), because of its advantage in fatigue monitoring, was preferred to PBRT for improving muscle strength for elite athletes in season. In contrast, higher resistance-trained males who performed PBRT slightly benefited maximal strength adaptation (Banyard et al., 2021). As the study’s participants were in the off-season, there was no intensive training program, so the benefit of monitoring fatigue was not fully utilized. Additionally, VBRT contained both load autoregulation and volume autoregulation, and it has been shown that the aforementioned various experimental designs significantly affected strength adaptations (Hickmott et al., 2022). For studies using simply load autoregulation (equal volume), the absence of velocity loss optimized resistance training programs resulted to training adaptations that were comparable to PBRT (Orange et al., 2019; Galiano et al., 2022), or even slightly better for the latter (Banyard et al., 2021). In controlled trials with a combination of load and volume autoregulation (non-equal volume), as in this study, LVP adjusted the load intensity to match velocity loss, and the VBRT group finished considerably less volume than the PBRT group (Peta, 2019; Dorrell et al., 2020; Ortega et al., 2020). Overall adaptation following VBRT was comparable to (Ortega et al., 2020) or superior to PBRT (Dorrell et al., 2020; Held et al., 2021). Indeed, this between-group difference in the induction effect (either squat or bench press) was subtle because these investigations were unable to identify a significant interaction effect (*p* > 0.05). Therefore, various statistical methods were used for between-group comparisons of VBRT and PBRT, including percentage differences (Dorrell et al., 2020), within-group effect sizes (Dorrell et al., 2020; Montalvo-Perez et al., 2021), standardized mean differences (Held et al., 2021), and magnitude-based inferences (Orange et al., 2019; Banyard et al., 2021). The following statistical methods were combined in the present research. The attitudes presented in the current study are somewhat comparable to those expressed in prior studies. In summary, the majority of VBRT *vs*. PBRT trial data appear to indicate a trend in which VBRT is not significantly different from PBRT in squat and bench press,. Up to this point, there has been no unified standard for the improvement effects of maximal strength between the two methods, and future studies should determine the explanation using a systematic review.

### Jump performance and upper limb explosiveness

These results confirmed the findings of a significant body of prior research on jump ability (Orange et al., 2019; Dorrell et al., 2020; Ortega et al., 2020; Banyard et al., 2021) and helped to partially clarified the mechanisms of adaptation to jump performance to some extent. Despite earlier research (Orange et al., 2019; Dorrell et al., 2020; Ortega et al., 2020; Banyard et al., 2021) focusing on CMJ height, it was found in these related studies that VBRT is more effective than PBRT at improving CMJ and DJ performance. According to prior research, heavy load resistance training mostly enhances SJ performance (Castro-Piñero et al., 2010), but not CMJ or DJ performance (Kubo et al., 2007). Specifically, resistance training programs and loads need to have targeted effects (Tsimahidis et al., 2010). Therefore, the intervention of this study involved 85% and 95% 1RM of heavy load training. In order to compare which of the two methods is more conducive to transforming explosive force adaptation and more suitable for basketball-specific performance, the height of CMJ, SJ and DJ as well as RSI were selected as the main outcome measures for a comprehensive comparison in this study. Based on maximal strength, reactive strength is to present the best muscle eccentric and concentric contraction speed (<200 m) with quick force, and the muscle shows a stretching-shorten cycle. In basketball, continuous jumping and upper limb confrontation often occur to fight for rebounds, hence DJ-RSI and PPU were selected as specific performance indicators. To our knowledge, no studies have compared eccentric utilization ratio and plyometric push-ups between both training methods. Both groups had a significant increase in DJ height (7.7% *vs.* 3.3%, ES = 0.48 to 0.31, ES = 0.48 to 0.31, respectively) with a significant time effect. According to previous evidence, the study of Orange et al. showed that VBRT improved CMJ height slightly better than PBRT (SMD = 0.53 to 0.4), and Dorrell et al. also obtained similar results (SMD = 0.23 to 0.06). And Banyard et al. explored differences in peak velocity of CMJ (7.4% *vs.* 4%, ES = 0.79 to 0.5). Additionally, a comparison of VL15 and VL30 velocity loss rates revealed that the gain of VL15 at CMJ height was significantly greater than that of VL30 (ES = 0.24–0.45). Notably, about the variable of DJ-RSI, there was a significant time effect and group by time interaction (*p* < 0.05), and the difference between groups was η_p_^2^ = 0.174. With the favorable improvement of VBRT for RSI and EUR/SSC%, it seemed to indicate that VBRT is more beneficial to improve the energy and power transfer efficiency of jumps. In addition, the SSC% increased from 2.8 to 8.1% (10% was best optimum). Previous literature has argued that Plyometric training tended to improve the results of SSC. The improvement in SSC% seems to explain a significant change in explosive force parameters induced by VBRT. In addition to load auto-regulation, monitoring and feedback technologies in VBRT seemed to be a significant contributory factor to the development of EUR, which could inspire the intention to accomplish concentric and eccentric phases during back squat training (Nagata et al., 2020). This PPU result was different from that of Bodden et al. (2019), who discovered no improvement in the plyometric push-up as a result of the acute effects of ballistic and non-ballistic bench press. According to Bodden et al., depending on the magnitude of the load applied, which resulted in acute fatigue and decreased the PPU characteristics, no significant improvement was made (Bodden et al., 2019). This was different from the findings discussed here since VBRT may be useful for monitoring acute fatigue and load autoregulation. This was different from the findings discussed here because VBRT can autoregulate load and monitoring acute fatigue. The chronic adaptation mechanism was helpful to understand that only VBRT induced a micro gain (*p* < 0.05) in upper limb power adaptation instead of PBRT, despite no time effect being observed. It was notable that there was a mutually reinforcing relationship between PPU performance and upper limb strength due to the post-activation potentiation (Wilcox et al., 2006). Which means that increasing the 1RM bench press with low-volume PPU before strength testing was equally successful (Wilcox et al., 2006; Krzysztofik and Wilk, 2020). Conditioning coaches should carefully consider this positive cycle mechanism to further optimize both short-term and long-term strength training programs. This finding had two potential explanations. These participants may have benefited from routine jump-focused basketball training, a lower-limb power-focused exercise that promoted the transfer of strength capacity to jump performance. Meanwhile, practitioners should be aware that the load prescription and adaptation mechanisms for the muscle groups in the lower limbs may be different from those in the upper limbs (Bartolomei et al., 2018). Taken together, and in conjunction with previous literature, the VBRT group was more effective than the PBRT group in improving jump height, jump velocity, EUR, PPU and DJ-RSI, and it appeared to be more suitable for developing basketball-specific performance. It could also be used as a reference for physical trainers and coaches in the future.

### Athletic and specific performance

In addition to the above comparisons about muscle strength and explosive power, this study also found differences in specific endurance performance, change of direction ability, sprint and standing long jump. The COD comparison results between the two groups are consistent with the previous work of Banyard et al. (2021), which reported similar results in the non-skilled legs change of direction ability test. As a new indicator for evaluating specific speed endurance, the results of 17-drill showed significant improvement only in the VBRT group (ES = −0.77 to −0.08), with similar results in the standing long jump. However, it should be highlighted that this contradicts three previous studies of men with resistance-trained males and rugby players (Orange et al., 2019; Ortega et al., 2020). The previous research indicated that VBRT was more effective than PBRT in developing short-distance sprint ability. However, sprint speeds of T10 and T20 in this study showed no intervention benefit, which could be attributed to the lack of plyometric and sprint training in this study.

Although Hexagon showed effective improvement and significant time effect, no group by time effect was found. Previous studies have indicated that training of these muscles can, among other things, enhance lower limb response and proprioception, thereby improving postural control. In the Hexagon test, there are two main efficacy parameters: a) rapid application of force when jumping into and out of the hexagon, and b) control of the kinematic results of explosive multi-directional lateral movements that disrupt the postural balance. It was evident that some of the exercises applied throughout the functional strength training directly stimulated proprioceptive quality in the knees, hips and trunk, enhancing postural control and thus improving hexagon test performance in the functional strength training (Tomljanović et al., 2011).

### Limitations and innovations

There are some limitations to this study, such as the short duration (6 weeks) and the low sample size. More importantly, this study used a randomized design to group participants, resulting in better baseline maximal strength for squat and bench press in the PBRT group than in the VBRT group (*p* > 0.05). Therefore, players with different strength levels experienced different increases in muscle strength and explosive power (Hoffman, 2011), which prevented us from drawing strong conclusions about the intervention effect of 1RM. However, analysis of covariance analysis were used to minimize the baseline imbalance of the above variables. Accordingly, larger sample size of randomized controlled trials are required to verify the observed differences in physical fitness indicators. In contrast, some innovations must also be acknowledged, first, in the middle of the 6-week intervention period, a mid-term test was added to verify dynamic changes in the individual 1RM and to further evaluate the changing trend of each indicator. Second, to ensure the effectiveness of participants’ training, this study employed the baseline FMS scores as the inclusion criteria, which, to our knowledge, is the first time FMS to be included as a criterion in a randomized controlled study.

## Conclusion

The current study shown that increases in muscular strength, Hexagon agility, and plyometric push-up were similar for both VBRT and PBRT methods. Additionally, VBRT appeared to be more effective at enhancing power adaptation and eliciting relevant athletic performance, with a favorable transfer effect from muscle strength to power, including vertical jump height and reactive strength, eccentric utilization ratio, direction-changing ability, and specific speed endurance. Finally, the real-time prescription loading approach employing MCV as a performance may provide heavier intensity than the traditional percentage-based fixed-load method depending on the state enhancement of the individual 1RM. Future research should examine if the EUR improvement brought about by VBRT is a result of the eccentric velocity of the back squat. Jump performance may be further studied using eccentric velocity monitoring in a velocity-based resistance training strategy.

### Practical applications

RT should optimize the conversion of training benefits into more athletic performances in addition to pursuing improvements in maximum strength. RT should contain as much detail as feasible, particularly with regard to movement patterns and contraction velocity. The findings revealed that VBRT seems to improve muscular coordination and be more focused than PBRT, with equivalent maximal strength increases, as demonstrated by improvements in CMJ, SJ, EUR, SSC%, DJ, DJ-RSI, COD, and 17-drill performance. Following a thorough analysis of above variables, VBRT is a superior approach for basketball players during the off-season.

## Data Availability

The original contributions presented in the study are included in the article/Supplementary material, further inquiries can be directed to the corresponding authors.

## References

[B1] Aagaard P. Simonsen E. B. Andersen J. L. Magnusson P. Dyhre-Poulsen P. (2002). Increased rate of force development and neural drive of human skeletal muscle following resistance training. J. Appl. Physiol. 93, 1318–1326. 10.1152/japplphysiol.00283.2002 12235031

[B2] Banyard H. G. Nosaka K. Vemon A. D. Haff G. G. (2018). The reliability of individualized load–velocity profiles. J. Exerc. Physiol. Online 13, 763–769. 10.1123/ijspp.2017-0610 29140148

[B3] Banyard H. G. Tufano J. J. Weakley J. J. S. Wu S. Jukic I. Nosaka K. (2021). Superior changes in jump, sprint, and change-of-direction performance but not maximal strength following 6 Weeks of velocity-based training compared with 1-repetition-maximum percentage-based training. Int. J. Sports Physiol. Perform. 16, 232–242. 10.1123/ijspp.2019-0999 32871553

[B4] Bartolomei S. Hoffman J. R. Stout J. R. Merni F. (2018). Effect of lower-body resistance training on upper-body strength adaptation in trained men. J. Strength Cond. Res. 32, 13–18. 10.1519/JSC.0000000000001639 29257792

[B5] Bodden D. Suchomel T. J. Lates A. Anagnost N. Moran M. F. Taber C. B. (2019). Acute effects of ballistic and non-ballistic bench press on plyometric push-up performance. Sports (Basel) 7, 47. 10.3390/sports7020047 30781654 PMC6409677

[B6] Bompa T. O. Buzzichelli C. (2019). Periodization-: theory and methodology of training. Champaign, IL: Human kinetics.

[B7] Buskard A. Zalma B. Cherup N. Armitage C. Dent C. Signorile J. F. (2018). Effects of linear periodization versus daily undulating periodization on neuromuscular performance and activities of daily living in an elderly population. Exp. Gerontol. 113, 199–208. 10.1016/j.exger.2018.09.029 30316811

[B8] Caldas L. C. Guimarães-Ferreira L. Duncan M. J. Leopoldo A. S. Leopoldo A. P. L. Lunz W. (2016). Traditional vs. undulating periodization in the context of muscular strength and hypertrophy: A meta-analysis. Int. J. Sports Sci. 6, 219–229. 10.5923/j.sports.20160606.04

[B9] Castagna C. Chaouachi A. Rampinini E. Chamari K. Impellizzeri F. (2009). Aerobic and explosive power performance of elite italian regional-level basketball players. J. Strength Cond. Res. 23, 1982–1987. 10.1519/JSC.0b013e3181b7f941 19855321

[B10] Castro-Piñero J. Ortega F. B. Artero E. G. Girela-Rejón M. J. Mora J. Sjöström M. (2010). Assessing muscular strength in youth: usefulness of standing long jump as a general index of muscular fitness. J. Strength Cond. Res. 24, 1810–1817. 10.1519/JSC.0b013e3181ddb03d 20555277

[B11] Chaouachi A. Brughelli M. Chamari K. Levin G. T. Ben Abdelkrim N. Laurencelle L. (2009). Lower limb maximal dynamic strength and agility determinants in elite basketball players. J. Strength Cond. Res. 23, 1570–1577. 10.1519/JSC.0b013e3181a4e7f0 19620905

[B12] Cohen J. (1988). Statistical power analysis for the behavioral sciences, 2nd Edn. Hillsdale, NJ: Lawrence Erlbaum.

[B13] De Hoyo M. Nunez F. J. Sanudo B. Gonzalo-Skok O. Munoz-Lopez A. Romero-Boza S. (2021). Predicting loading intensity measuring velocity in barbell hip thrust exercise. J. Strength Cond. Res. 35, 2075–2081. 10.1519/JSC.0000000000003159 31009439

[B14] Dorrell H. F. Moore J. M. Gee T. I. (2020). Comparison of individual and group-based load-velocity profiling as a means to dictate training load over a 6-week strength and power intervention. J. Sport Sci. 38, 2013–2020. 10.1080/02640414.2020.1767338 32516094

[B15] Doyle T. (2005). How effectively is the stretch-shortening cycle being used by athletes? Strength Cond. Coach 13, 7–12.

[B16] Galiano C. Pareja-Blanco F. Hidalgo De Mora J. Saez De Villarreal E. (2022). Low-velocity loss induces similar strength gains to moderate-velocity loss during resistance training. J. Strength Cond. Res. 36, 340–345. 10.1519/JSC.0000000000003487 31904715

[B17] González-Badillo J. J. Sánchez-Medina L. (2010). Movement velocity as a measure of loading intensity in resistance training. Int. J. Sports Med. 31, 347–352. 10.1055/s-0030-1248333 20180176

[B18] González-Badillo J. J. Marques M. C. Sánchez-Medina L. (2011). The importance of movement velocity as a measure to control resistance training intensity. J. Hum. Kinet. 29, 15–19. 10.2478/v10078-011-0053-6 23487504 PMC3588891

[B19] González-Badillo J. J. Yañez-García J. M. Mora-Custodio R. Rodríguez-Rosell D. (2017). Velocity loss as a variable for monitoring resistance exercise. Int. J. Sports Med. 38, 217–225. 10.1055/s-0042-120324 28192832

[B20] Greig L. Stephens Hemingway B. H. Aspe R. R. Cooper K. Comfort P. Swinton P. A. (2020). Autoregulation in resistance training: Addressing the inconsistencies. Sports Med. 50, 1873–1887. 10.1007/s40279-020-01330-8 32813181 PMC7575491

[B21] Gribble P. A. Hertel J. Plisky P. (2012). Using the star excursion balance test to assess dynamic postural-control deficits and outcomes in lower extremity injury: A literature and systematic review. J. Athl. Train. 47, 339–357. 10.4085/1062-6050-47.3.08 22892416 PMC3392165

[B22] Hawkins S. B. Doyle T. L. Mcguigan M. R. (2009). The effect of different training programs on eccentric energy utilization in college-aged males. J. Strength Cond. Res. 23, 1996–2002. 10.1519/JSC.0b013e3181b3dd57 19855323

[B23] Held S. Hecksteden A. Meyer T. Donath L. (2021). Improved strength and recovery after velocity-based training: A randomized controlled trial. Int. J. Sports Physiol. Perform. 16, 1185–1193. 10.1123/ijspp.2020-0451 33547265

[B24] Helms E. R. Brown S. R. Cross M. R. Storey A. Cronin J. Zourdos M. C. (2017). Self-rated accuracy of rating of perceived exertion-based load prescription in powerlifters. J. Strength Cond. Res. 31, 2938–2943. 10.1519/JSC.0000000000002097 28933716

[B25] Hickmott L. M. Chilibeck P. D. Shaw K. A. Butcher S. J. (2022). The effect of load and volume autoregulation on muscular strength and hypertrophy: A systematic review and meta-analysis. Sports Medicine-Open 8, 9–35. 10.1186/s40798-021-00404-9 35038063 PMC8762534

[B26] Hoffman J. (2011). NSCA's guide to program design. Champaign, IL: Human Kinetics.

[B27] Hopkins W. G. Marshall S. W. Batterham A. M. Hanin J. (2009). Progressive statistics for studies in sports medicine and exercise science. Med. Sci. Sports Exerc. 41, 3–13. 10.1249/MSS.0b013e31818cb278 19092709

[B28] Hopkins W. G. (2006). Spreadsheets for analysis of controlled trials, with adjustment for a subject characteristic. Sport Sci. 10, 46–50.

[B29] Hopkins W. G. (2017). Spreadsheets for analysis of controlled trials, crossovers and time series. Sports Sci. 21, 1–5.

[B30] Inoue D. S. De Mello M. T. Foschini D. Lira F. S. Ganen A. D. P. Campos R. M. D. S. (2015). Linear and undulating periodized strength plus aerobic training promote similar benefits and lead to improvement of insulin resistance on obese adolescents. J. Diabetes Complicat. 29, 258–264. 10.1016/j.jdiacomp.2014.11.002 25441178

[B31] Izquierdo M. Gonzalez-Badillo J. J. Hakkinen K. Ibanez J. Kraemer W. J. Altadill A. (2006). Effect of loading on unintentional lifting velocity declines during single sets of repetitions to failure during upper and lower extremity muscle actions. Int. J. Sports Med. 27, 718–724. 10.1055/s-2005-872825 16944400

[B32] Jovanović M. Flanagan E. P. (2014). Researched applications of velocity based strength training. J. Aust. Strength Cond. 22, 58–69.

[B33] Jukic I. Castilla A. P. Ramos A. G. Van Hooren B. Mcguigan M. R. Helms E. R. (2022). The acute and chronic effects of implementing velocity loss thresholds during resistance training: A systematic review, meta-analysis, and critical evaluation of the literature. Sports Med., 1–38. 10.1007/s40279-022-01754-4 PMC9807551 36178597

[B34] Kraemer W. J. Ratamess N. A. (2004). Fundamentals of resistance training: Progression and exercise prescription. Med. Sci. Sports Exerc. 36, 674–688. 10.1249/01.mss.0000121945.36635.61 15064596

[B35] Kraus K. Schutz E. Taylor W. R. Doyscher R. (2014). Efficacy of the functional movement screen: A review. J. Strength Cond. Res. 28, 3571–3584. 10.1519/JSC.0000000000000556 24918299

[B36] Krzysztofik M. Wilk M. (2020). The effects of plyometric conditioning on post-activation bench press performance. J. Hum. Kinet. 74, 99–108. 10.2478/hukin-2020-0017 33312279 PMC7706649

[B37] Kubo K. Morimoto M. Komuro T. Yata H. Tsunoda N. Kanehisa H. (2007). Effects of plyometric and weight training on muscle-tendon complex and jump performance. Med. Sci. Sports Exerc. 39, 1801–1810. 10.1249/mss.0b013e31813e630a 17909408

[B38] Larsen S. Kristiansen E. Van Den Tillaar R. (2021). Effects of subjective and objective autoregulation methods for intensity and volume on enhancing maximal strength during resistance-training interventions: A systematic review. PeerJ 9, e10663. 10.7717/peerj.10663 33520457 PMC7810043

[B39] Liao K. F. Wang X. X. Han M. Y. Li L. L. Nassis G. P. Li Y. M. (2021). Effects of velocity based training vs. traditional 1RM percentage-based training on improving strength, jump, linear sprint and change of direction speed performance: A systematic review with meta-analysis. PLoS One 16, e0259790. 10.1371/journal.pone.0259790 34793506 PMC8601436

[B40] Mann J. B. Thyfault J. P. Ivey P. A. Sayers S. P. (2010). The effect of autoregulatory progressive resistance exercise vs. linear periodization on strength improvement in college athletes. J. Strength Cond. Res. 24, 1718–1723. 10.1519/JSC.0b013e3181def4a6 20543732

[B41] Mann J. B. Ivey P. A. Sayers S. P. (2015). Velocity-based training in football. Strength Cond. J. 37, 52–57. 10.1519/ssc.0000000000000177

[B42] Montalvo-Perez A. Alejo L. B. Valenzuela P. L. Gil-Cabrera J. Talavera E. Luia A. (2021). Traditional versus velocity-based resistance training in competitive female cyclists: A randomized controlled trial. Front. Physiol. 12, 586113. 10.3389/fphys.2021.586113 33716761 PMC7947617

[B43] Nagata A. Doma K. Yamashita D. Hasegawa H. Mori S. (2020). The effect of augmented feedback type and frequency on velocity-based training-induced adaptation and retention. J. Strength Cond. Res. 34, 3110–3117. 10.1519/JSC.0000000000002514 33105361

[B44] Orange S. T. Metcalfe J. W. Robinson A. Applegarth M. J. Liefeith A. (2019). Effects of in-season velocity- versus percentage-based training in academy rugby league players. Int. J. Sports Physiol. Perform. 15, 554–561. 10.1123/ijspp.2019-0058 31672928

[B45] Orange S. T. Metcalfe J. W. Marshall P. Vince R. V. Madden L. A. Liefeith A. (2020). Test-retest reliability of a commercial linear position transducer (GymAware PowerTool) to measure velocity and power in the back squat and bench press. J. Strength Cond. Res. 34, 728–737. 10.1519/JSC.0000000000002715 29952868

[B46] Orange S. T. Hritz A. Pearson L. Jeffries O. Jones T. W. Steele J. (2022). Comparison of the effects of velocity-based vs. traditional resistance training methods on adaptations in strength, power, and sprint speed: A systematic review, meta-analysis, and quality of evidence appraisal. J. Sports Sci. 40, 1220–1234. 10.1080/02640414.2022.2059320 35380511

[B47] Ortega J. a. F. Reyes Y. G. D. L. Peña F. R. G. (2020). Effects of strength training based on velocity versus traditional training on muscle mass, neuromuscular activation, and indicators of maximal power and strength in girls soccer players. Apunt. Med. l'Esport 55, 53–61. 10.1016/j.apunsm.2020.03.002

[B48] Pareja-Blanco F. Rodriguez-Rosell D. Sanchez-Medina L. Sanchis-Moysi J. Dorado C. Mora-Custodio R. (2017). Effects of velocity loss during resistance training on athletic performance, strength gains and muscle adaptations. Scand. J. Med. Sci. Sports 27, 724–735. 10.1111/sms.12678 27038416

[B49] Peta H. (2019). The influence of velocity-based resistance training on strength and power development. Hamilton: The University of Waikato.

[B50] Poliquin C. (1988). FOOTBALL: Five steps to increasing the effectiveness of your strength training program. NSCA J. 10, 34–39. 10.1519/0744-0049(1988)010<0034:fstite>2.3.co;2

[B51] Ransone J. (2016). “Physiologic profile of basketball athletes,” in Nutrition & recovery needs of the basketball athlete, 10.

[B52] Rice P. E. Goodman C. L. Capps C. R. Triplett N. T. Erickson T. M. Mcbride J. M. (2017). Force–and power–time curve comparison during jumping between strength-matched male and female basketball players. Eur. J. Sport Sci. 17, 286–293. 10.1080/17461391.2016.1236840 27691454

[B53] Richens B. Cleather D. J. (2014). The relationship between the number of repetitions performed at given intensities is different in endurance and strength trained athletes. Biol. Sport 31, 157–161. 10.5604/20831862.1099047 24899782 PMC4042664

[B54] Robertson R. J. Goss F. L. Rutkowski J. Lenz B. Dixon C. Timmer J. (2003). Concurrent validation of the OMNI perceived exertion scale for resistance exercise. Med. Sci. Sports Exerc. 35, 333–341. 10.1249/01.MSS.0000048831.15016.2A 12569225

[B55] Ruther C. L. Golden C. L. Harris R. T. Dudley G. A. (1995). Hypertrophy, resistance training, and the nature of skeletal muscle activation. J. Strength Cond. Res. 9, 155–159. 10.1519/1533-4287(1995)009<0155:hrtatn>2.3.co;2

[B56] Sabin S. I. Alexandru S. D. (2015). Testing agility and balance in volleyball game. Buchares: National UniversIty of Physical Education and Sport.

[B57] Şahin M. D. Aybek E. C. (2019). Jamovi: An easy to use statistical software for the social scientists. Int J Assess. Tools Educ 6, 670–692. 10.21449/ijate.661803

[B58] Sander A. Keiner M. Wirth K. Schmidtbleicher D. (2013). Influence of a 2-year strength training programme on power performance in elite youth soccer players. Eur. J. Sport Sci. 13, 445–451. 10.1080/17461391.2012.742572 24050460

[B59] Santos E. J. Janeira M. A. (2012). The effects of resistance training on explosive strength indicators in adolescent basketball players. J. Strength Cond. Res. 26, 2641–2647. 10.1519/JSC.0b013e31823f8dd4 22108528

[B60] Simenz C. J. Dugan C. A. Ebben W. P. (2005). Strength and conditioning practices of National Basketball Association strength and conditioning coaches. J. Strength Cond. Res. 19, 495–504. 10.1519/15264.1 16095396

[B61] Spiteri T. Newton R. U. Binetti M. Hart N. H. Sheppard J. M. Nimphius S. (2015). Mechanical determinants of faster change of direction and agility performance in female basketball athletes. J. Strength Cond. Res. 29, 2205–2214. 10.1519/JSC.0000000000000876 25734779

[B62] Stewart P. F. Turner A. N. Miller S. C. (2014). Reliability, factorial validity, and interrelationships of five commonly used change of direction speed tests. Scand. J. Med. Sci. Sports 24, 500–506. 10.1111/sms.12019 23176602

[B63] Tomljanović M. Spasić M. Gabrilo G. Uljević O. Foretić N. (2011). Effects of five weeks of functional vs. traditional resistance training on anthropometric and motor performance variables. Kinesiology 43, 145–154.

[B64] Tsimahidis K. Galazoulas C. Skoufas D. Papaiakovou G. Bassa E. Patikas D. (2010). The effect of sprinting after each set of heavy resistance training on the running speed and jumping performance of young basketball players. J. Strength Cond. Res. 24, 2102–2108. 10.1519/JSC.0b013e3181e2e1ed 20613645

[B65] Vickers A. J. Altman D. G. (2001). Statistics notes: Analysing controlled trials with baseline and follow up measurements. BMJ 323, 1123–1124. 10.1136/bmj.323.7321.1123 11701584 PMC1121605

[B66] Weakley J. Mann B. Banyard H. Mclaren S. Garcia-Ramos A. (2020). Velocity-based training: From theory to application. Strength Cond. J. 43, 31–49. 10.1519/ssc.0000000000000560

[B67] Weakley J. Morrison M. Garcia-Ramos A. Johnston R. James L. Cole M. H. (2021). The validity and reliability of commercially available resistance training monitoring devices: A systematic review. Sports Med. 51, 443–502. 10.1007/s40279-020-01382-w 33475985 PMC7900050

[B68] Wilcox J. Larson R. Brochu K. M. Faigenbaum A. D. (2006). Acute explosive-force movements enhance bench-press performance in athletic men. Int. J. Sports Physiol. Perform. 1, 261–269. 10.1123/ijspp.1.3.261 19116439

[B69] Zhang M. Tan Q. Sun J. Ding S. Yang Q. Zhang Z. (2022). Comparison of velocity and percentage-based training on maximal strength: Meta-analysis. Int. J. Sports Med. 43, 981–995. 10.1055/a-1790-8546 35255509

[B70] Zhihui W. (2020). The effect of velocity-based strength training on the lower limbs power-related athletic abilities of college basketball athletes by back squat. Wuhan: Wuhan Sports University.

